# Acute and Chronic Exercise Effects on Human Memory: What We Know and Where to Go from Here

**DOI:** 10.3390/jcm10214812

**Published:** 2021-10-20

**Authors:** Paul D. Loprinzi, Marc Roig, Jennifer L. Etnier, Phillip D. Tomporowski, Michelle Voss

**Affiliations:** 1 Exercise and Memory Laboratory, Department of Health, Exercise Science and Recreation Management, University of Mississippi, Oxford, MS 38677, USA; 2 Memory and Motor Rehabilitation Laboratory (MEMORY-LAB), School of Physical and Occupational Therapy, Faculty of Medicine, McGill University, Montreal, QC H3A 0G4, Canada; marc.roigpull@mcgill.ca; 3 Physical Activity and Cognition Laboratory, Department of Kinesiology, University of North Carolina at Greensboro, Greensboro, NC 27412, USA; jletnier@uncg.edu; 4 Cognition and Skill Acquisition Laboratory, Department of Kinesiology, University of Georgia, Athens, GA 30602, USA; ptomporo@uga.edu; 5 Health, Brain & Cognition Laboratory, Department of Psychological and Brain Sciences, University of Iowa, Iowa City, IA 52242, USA; michelle-voss@uiowa.edu

## 1. Memory Systems

Although the acquisition, storage, and retrieval of memories was once thought to happen within a single memory system with multiple processes operating on it, it is now believed that memory is comprised of both distinct and interacting brain systems [1,2]. Declarative memory is representational, involving the conscious recollection of facts (semantic) and events (episodic). Nondeclarative memory is dispositional and expressed through performance, not recollection, including habits, skills, priming, and simplistic forms of conditioning. An important principle of declarative memory is the detection and encoding of a single event, occurring at a particular time and place, whereas nondeclarative memory involves the gradual extraction of common elements from a series of separate events [2]. 

Long-term declarative memory is often characterized into several phases, including acquisition (encoding and consolidation), storage, and retrieval. Encoding, which can occur consciously or subconsciously/unconsciously, involves the processing of information (e.g., internal thoughts, external stimuli) to be stored for later retrieval. From a neurophysiological perspective, information encoding creates a memory trace that is comprised of a neural network (engram) representing the memory. Memory consolidation, or the processes in which the memory trace becomes stabilized, is thought to occur at multiple levels, including the cellular/synaptic level (i.e., synaptic consolidation) and the brain system level (i.e., system consolidation, in which the trace is distributed from the hippocampus to neocortical areas for long-term storage) [3]; for an alternative account to system consolidation, the reader is referred to other work discussing contextual binding theory [4]. Once stabilized, the memory is “stored”, possibly within the dendritic spines [5]. Lastly, upon the use of a cue (e.g., internal mental state or external stimuli), or when occurring spontaneously, memories can be retrieved and, subsequently, enter a state of reconsolidation [6]. 

A fundamental distinction is made between short-term (inclusive of working memory) and long-term memory. Short-term memory refers to the relatively temporary storage of information. Without rehearsal, short-term memory is brief (e.g., 15–30 s) [7], but this duration is heavily influenced by, for example, the type of stimuli that is to be remembered (e.g., words vs. pictures), the cognitive load of the stimuli (e.g., three vs. seven digits), the type of memory task (e.g., free recall vs. recognition), and the degree of interference (e.g., proactive vs. no interference). Related to short-term memory, working memory, however, engages brain structures and processes involved in the manipulation of content, rather than just the maintenance of that content over a particular duration. Thus, working memory requires actively manipulating the content of memories in the service of directing attention towards what is most relevant for current behavior and other aspects of cognition. These interactions between memory and selective attention are why working memory is often considered to be one domain of executive functioning. Importantly, long-term memory may start to form shortly after acquisition [8]. In contrast to short-term memory, long-term memory processes result in a more durable memory trace with a much larger capacity than both short-term and working memory. 

We will now discuss the effects of acute exercise (a single bout of exercise) on memory, with a focus on aerobic exercise because it predominates the literature. Following this, we will discuss the effects of chronic exercise (repeated bouts of exercise) on memory.

As shown in the figure, and depending on the memory phase or mechanism that is targeted, the timing of acute exercise relative to the memory task may vary, with acute exercise typically occurring before or after the memory task (i.e., encoding). For example, for a long-term memory assessment involving the retrospective recall of information, acute exercise may occur prior to learning the stimuli in order to evaluate encoding-based mechanisms. In contrast, acute exercise may occur after encoding the stimuli to evaluate the effects of exercise on consolidation-based aspects. Although not the central focus of the present paper, a detailed discussion on how memory is influenced during exercise can be found in a review by Tomporowski and Qazi [9]. 

## 2. Effects of Acute Exercise on Memory

McMorris previously detailed the history of research evaluating the effects of acute exercise on cognition [10]. The following narrative highlights several historical reviews detailing these effects. In a 1986 narrative review, Tomporowski and Ellis [11] suggested that acute exercise had minimal effects on improving post-exercise memory. Similar null results were obtained in a 1997 quantitative review by Etnier et al. [12]. Extending these prior reviews, a 2003 narrative review by Tomporowski suggested that acute exercise may improve specific aspects of informational processing, but it still appeared to have minimal effects on post-exercise memory function [13]. In a 2012 quantitative review, Chang et al. [14] also showed null effects for post-exercise memory, but a different story unfolded when evaluating distinct memory systems across exercise paradigms (i.e., including memory during exercise and memory after exercise). When considered across all exercise paradigms, acute exercise improved memory for free recall and visual short-term memory but not other types, such as memory for sequences. In a 2013 quantitative review exclusively focused on memory, Roig et al. [15] demonstrated evidence that both short- (ES = 0.26) and long-term memory (ES = 0.52) can be enhanced after a bout of acute exercise. A 2019 quantitative review by Loprinzi et al. [16] showed that acute exercise can improve memory even when occurring before memory encoding or during memory consolidation. Finally, in a 2020 narrative review, Blomstrand and Engvall [17] concurred with the latest reviews [14,15,16], showing that acute exercise can improve memory function. Notably, empirical evidence suggests that these beneficial effects occur across the lifespan, including in children [18,19], young adults [20,21,22], and older adults [23]. Collectively, these reviews suggest that, from the most recent experimental work on this topic, utilizing more robust designs and considering potential moderators that have been shown to influence effect sizes (e.g., memory type and timing of acute exercise in reference to the memory task [24]), acute exercise may be effective in enhancing post-exercise memory. A further discussion about potential moderators of the acute exercise–memory relationship will be discussed in a later section.

## 3. Effects of Chronic Exercise on Memory

Etnier et al. [25] previously detailed the history of research evaluating the effects of chronic exercise on cognition. In a 1997 quantitative review, Etnier et al. [12] reported a beneficial effect of chronic exercise engagement on memory function (ES = 0.45), an effect that was substantially greater than the relationship between acute exercise and memory (ES = 0.10). When evaluating short- and long-term memory separately, Roig et al. [15] showed that chronic exercise was not effective in enhancing long-term memory (ES = 0.07), but it improved short-term memory (ES = 0.15). Similar results were demonstrated in a 2017 quantitative review by Rathore and Lom [26], showing that chronic exercise was effective in enhancing short-term and working memory performances.

## 4. Selective Use of Study Design to Target Memory Phases and Exercise Mechanisms

As demonstrated, evidence suggests that both acute and chronic exercise can improve memory function. The appropriate design and use of acute and chronic exercise interventions can provide critical insight as to when and how exercise improves memory for different types of information. Acute paradigms are ideally suited to study time-locked interactions between exercise and the specific phases of memory. This paradigm is also ideal to study transient neuroplasticity changes involved in memory processes. Chronic paradigms, in contrast, are less well-suited to study time-locked interactions but are better equipped to study long-term adaptations (e.g., changes in the hippocampal volume). Notably, both paradigms can be combined. The figure illustrates the use of acute and chronic exercise designs to evaluate their memorial effects across different phases of memory. 

**Figure 1:**
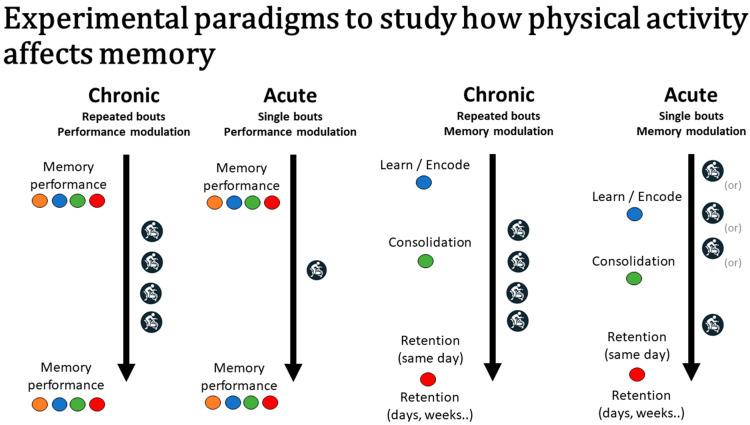
Illustration of the use of acute and chronic exercise designs to evaluate the effects of exercise on memory and across different phases of memory. Performance modulation refers to changes in memory performance without focusing on a specific memory phase. In contrast, memory modulation refers to evaluating how a long-term memory is influenced from modulating a particular phase of memory. For performance modulation, exercise, either repeated bouts (chronic exercise) or a single bout (acute exercise), would occur between a memory test that included both exposure and retrieval. For memory modulation, repeated bouts of exercise (chronic exercise) would typically occur during the consolidation period, whereas, for acute exercise, the bout of exercise may occur at any phase of memory. Blue circles represent encoding; green, consolidation; and red, retrieval. Orange circles represent short-term memory (inclusive of working memory).

As illustrated in Figure 1, and based on when the bout of exercise is performed relative to specific memory processes, different memory processes and mechanisms can be evaluated. To evaluate the effects of acute exercise on encoding-related mechanisms, the bout of exercise should occur prior to memory encoding, with memory retrieval occurring shortly after encoding, to better isolate encoding (rather than consolidation) mechanisms. The use of distractor items between encoding and retrieval can also help to address concerns about rehearsal when long-term memory is of interest, although potential interferences in the memory process need to be considered. Potential encoding-related mechanisms may include, for example, enhanced attention, increased production and release of neurotransmitters, and the priming of neural networks that represent the memory. For example, potential exercise-induced increases in attention may help to increase the memory performance by altering monitoring processes at encoding and retrieval. Exercise may also increase the production and release of select proteins (e.g., BDNF—brain-derived neurotrophic factor) and activate select receptors (N-Methyl-D-aspartate) to increase the strengthening of neural networks that provide a neural representation of the memory. 

To isolate the effects of acute exercise on consolidation-related mechanisms, the bout of exercise could be performed at some point after encoding but during the memory consolidation window and not too close to memory retrieval. Mechanistically, the bout of acute exercise is expected to increase the production of neurotrophins and growth factors to stabilize the memory trace. For example, exercise-induced CREB (cAMP response element-binding) phosphorylation may increase the transcription of key regulatory proteins to stabilize the memory trace. Relatedly, potential exercise-induced increases in PSD-95 (postsynaptic density protein 95) may help induce receptor incorporation (e.g., α-amino-3-hydroxy-5-methyl-4-isoxazolepropionic acid; AMPA) and synapse stabilization. Interestingly, and as hypothesized previously [27] and tested recently [28], exercise-induced activation of the endocannabinoid system may play an important role in altering mediators (e.g., BDNF and hippocampal cell proliferation) of the exercise–memory relationship. 

To isolate the effects of acute exercise on retrieval-related mechanisms, the bout of exercise would typically occur after encoding, after an appropriate period of time for consolidation, and shortly before memory retrieval. A potential mechanism through which acute exercise may alter retrieval-related mechanisms is through improvements in item-invariant retrieval, which involves the executive control to allocate mental resources to search for items in memory. Speculatively, exercise may help to increase such executive control-related monitoring processes to facilitate successful memory retrieval [29]. 

For a more detailed review of these potential encoding, consolidation, and retrieval-related mechanisms, the reader is referred elsewhere [30,31,32,33,34,35,36,37,38,39]. Importantly, we need to perform additional mechanistic studies in humans, as there is inconsistency in the literature regarding the veracity and translational aspects of these neurophysiological mechanisms [40,41,42,43], as well as limited research directly evaluating some of the encoding (e.g., attention) and retrieval-based mechanisms. As such, future mechanistic work across multiple fields, such as exercise science, psychology (e.g., attentional, monitoring, and decisional processes), and cognitive neuroscience (e.g., neurophysiological), are needed. 

As shown in the figure, during chronic training, multiple bouts of exercise are performed. When focusing on changes in memory performance, the repeated bouts of exercise may occur between a memory test, including both encoding and retrieval. In contrast, when focused on the modulation of a specific memory process (i.e., memory modulation), the repeated bouts of exercise may occur at different phases of forming new long-term memories. Often-evaluated mechanisms of chronic exercise on memory include, for example, increased brain capillarization and perfusion in regions important for long-term memory, such as the hippocampus, and increased spine density and neurogenesis in the hippocampus (e.g., see recent work on the exercise-related liver-to-brain axis to induce hippocampal neurogenesis and improved memory [32,44]), which may help to increase the communication and functional connectivity (i.e., synchrony of functional activity in non-adjacent brain regions) within and across neural networks. Notably, recent works suggest that alterations in functional connectivity may account for some of the exercise-induced improvements in cognitive function [45,46]. Details on how chronic exercise can influence neurotransmitter systems, neurotrophic stimulation, and cerebral circulation can be found elsewhere [47,48,49,50].

## 5. Controversies and Inconsistencies: Discussion of Moderators

Although both acute and chronic exercise can improve memory, there are numerous factors that may moderate these effects, including aspects related to the individual, dimensions of exercise, type of memory, and other design-related characteristics. 

***Acute Exercise. Individual Factors***. Although not an exhaustive list, various individual-level factors may influence the exercise–memory relationship, such as age, biological sex, cardiorespiratory fitness, individual differences in memory function, and BDNF genotype; for other factors to consider, see work by Herold et al. [51]. Age appears to be an important moderator of the effects of exercise on memory. Recent work suggests that acute exercise can improve memory in older adults [23], and, for select memory systems (e.g., working memory), acute exercise may improve memory for individuals across the adult lifespan [52]. Meta-analytic research suggests that the effect of acute exercise on short-term memory and working memory may be more pronounced for younger adults [15,16]; however, the small number of studies conducted with middle-aged and older adults suggests that additional empirical work is necessary. 

Regarding biological sex, females tend to outperform males in most memory tasks [53], but minimal research has evaluated whether acute exercise influences memory differentially for females and males [54], with some research suggesting that benefits may be in favor of females [54,55,56]. In addition to age and biological sex, it is possible that an individual’s cardiorespiratory fitness may moderate the physiological demands of acute exercise and/or perceptions of the exercise and, in turn, influence the effects of exercise on memory [33]. It is also possible that fitness may moderate the effects of acute exercise on putative underlying mechanisms, such as BDNF. Szuhany et al. [57] reported a larger average increase in BDNF (g = 0.59) in response to acute exercise following a training program (3–24 weeks) as compared to before the training program [58]. Meta-analytic findings show that, while cardiorespiratory fitness did not influence the effects of acute exercise on short-term memory, individuals with average cardiorespiratory fitness showed greater exercise-induced effects on long-term memory [15]. This finding, however, should be interpreted with caution, as a single study was included in this specific moderation analysis.

An individual’s baseline memory function is thought to potentially influence the acute exercise–memory relationship [59,60]. In particular, these two studies showed that the benefits from acute exercise were larger for those with lower baseline performance for a measure of working memory [60] and spatial memory [59]. Further work is needed to ensure these effects are not driven by a statistical regression-to-the-mean effect. It may also be worth evaluating whether the relationship between age and individual differences influences the memorial response to exercise. For example, potential effects for older adults may be due to their lower cognitive function.

Lastly, the neurotrophin, BDNF, has been suggested to be a critical protein involved in memory function and, by extension, the relationship between acute exercise and memory [61]. As previously mentioned, there is evidence that BDNF may play a role in explaining the combined impact of chronic training and acute exercise. However, it is important to remember that we are still relatively naïve as to the role of BDNF in this relationship. In humans, there is inconsistent evidence that this protein [41,62], or the gene that encodes this protein [63], influences the effects of acute exercise on memory. Furthermore, the specific role of BDNF in explaining the effects of exercise on memory are complicated by the fact that it can only be measured peripherally in humans, so that inferences about the central levels of BNDF are speculative. Additionally, methodological variations (across studies) in the source for BDNF analysis (serum vs. plasma), storage time, and centrifuge strategy can have pronounced effects on BDNF levels and its association with exercise/fitness [64]. Lastly, future research will benefit from conducting assays of BDNF that allow for a distinction between pro and mature isoforms because of their differing roles relative to cellular models of memory [61,65].

***Dimensions of Exercise***. Meta-analytic findings suggest that shorter exercise durations (<20 min) may be optimal in enhancing short-term memory, whereas short- and medium-duration acute exercise (<40 min) may better influence long-term memory function [15]. Regarding exercise intensity, moderate-intensity acute exercise may better serve working memory performances [66], whereas high-intensity acute exercise may be optimal for lower-order memory tasks (e.g., list-learning paradigms) [16]. Even maximal-intensity exercise may provide superior benefits for long-term memory function [41]. There are conflicting findings, however, as some research suggests that moderate-intensity acute exercise may be optimal in enhancing memory [67,68]. In a meta-analysis of cognition in general (i.e., not specific to memory), the findings from Chang et al. [14] show that the optimal exercise intensity may differ depending on how long post-exercise the cognitive measure is assessed. When cognition was assessed immediately after exercise, very light, light, and moderate intensity had benefits, but hard, very hard, and maximal exercise did not have positive effects. However, when cognition was assessed after a delay, very light was no longer beneficial, but hard and very hard had positive effects. These findings, which need to be evaluated in the memory domain, suggest an important interaction between exercise intensity and post-exercise recovery period in influencing cognitive performance.

The timing of the bout of exercise relative to the memory task is an important factor influencing the exercise–memory relationship [24]. Research demonstrates that acute exercise (compared to no exercise) prior to memory encoding can enhance long-term memory [15,20,21,23,69,70,71], and similarly, long-term memory can be enhanced when exercise occurs post-encoding [29,56,72]. Further, some research demonstrates that the memory benefits from exercise occur regardless of whether acute exercise occurs before or after memory encoding [29,73], and one study even suggests that exercise performed both before and after memory encoding results in the largest benefits for long-term memory [22].

The modality of exercise is another dimension of exercise that is worth considering. Meta-analytic research suggests that walking may optimize short-term memory [15], whereas cycling may have a greater benefit for long-term memory improvement [15,16]. Further, the movement pattern (e.g., simple predictable movement skills compared to more complex, unpredictable movement patterns) during ambulation may also have a unique effect on memory function [74,75]. Relatedly, the degree of cognitive engagement during ambulation may also provide interesting insights into this topic. More research is needed to explain the effects of different modalities of exercise on memory.

***Memory Type***. In general, acute exercise seems to have a more favorable effect on improving long-term memory compared to short-term memory, but the potential confounding effects of fatigue in cognitive exercise studies is important to consider [76], but may be difficult to control. Given that short-term memory performance is likely to influence long-term memory performance, and given that acute exercise has been shown to enhance short-term memory, at this point, it is not entirely clear if the long-term memory gains observed after exercise are driven by its effects on short-term memory. Labban and Etnier [20] conducted a study using repeated exposures to a word list without any short-term recall and still demonstrated benefits to long-term memory in response to acute exercise performed prior to the word list exposure. However, there is still much to learn, as it is not clear if the observed improvements in long-term memory from exercise are due to processes occurring after learning and short-term memory retention (memory consolidation), or if they are rather driven by exercise-induced improvements in learning and short-term memory [77,78]. As such, future work should consider evaluating the effects of acute exercise on long-term memory with and without the presence of a short-term (or earlier) memory assessment. 

In addition, acute exercise may have a differential effect on distinct memory systems across the longevity of the memory (short vs. long). There is some evidence to suggest that acute exercise favors visuospatial memory for short-term memories, whereas acute exercise more strongly benefits verbal–auditory and procedural long-term memory [15]. Interestingly, research has also started to explore the effects of acute exercise on other distinct memory types, such as implicit memory [79,80], emotional memory [81,82,83], and false memory [67,84,85]. We also need additional exercise and memory research that comprehensively evaluates and integrates each of the constituents of episodic memory, namely the “what”, “where”, and “when” aspects of episodes [86].

***Protocol Considerations***. Various aspects of the study protocol could potentially moderate the acute exercise–memory relationship. For example, variations in the way in which the exercise intensity is prescribed (e.g., based on the estimated or measured heart rate max, ventilatory or lactate threshold, heart rate reserve or VO_2_ reserve, or brain-derived parameters) can play an important role in the accuracy of the prescribed exercise intensity and, by extension, the conclusions rendered regarding the relationship between exercise intensity and memory. As such, the method used to determine the intensity of exercise should be carefully considered. For specific recommendations, the reader is referred elsewhere [51,87,88]. 

Aspects of the memory protocol should also be carefully considered. If the retention interval, or the period between encoding and retrieval, is too short, then potential exercise-related changes in memory consolidation may not have enough time to occur. Relatedly, if the retention interval is too long, floor effects in memory performances may occur. Additionally, if too many memory assessments (e.g., immediate memory recall and 10-min delayed recall) occur within a relatively short period of time, this may potentially mask any exercise-related effects on memory or increase the risk of relearning or reconsolidation, which could affect the memory outcome. Further, the initial memory assessment (e.g., immediate recall) may potentially influence the ability of acute exercise to enhance long-term memory. This aligns with meta-analytic research showing that implementing a retention test long after the end of the exercise bout maximizes the effects of acute exercise on long-term memory [15]. Thus, the timing of the memory task should be carefully considered based on whether the goal is to evaluate encoding, consolidation, or retrieval effects from exercise. 

Additional protocol considerations include the type of processing during memory encoding. Without proper control of this, participants may engage in a variety of different types of processing, which may, in part, be responsible for the mixed findings in the literature. Increasing the likelihood that all participants process the stimuli similarly in a single study may help to produce more robust findings. For example, shallow levels of processing could involve having all the participants read aloud each word/stimulus, which may help ensure that all participants, to some extent, are attending to the stimuli. Alternatively, orienting tasks (e.g., sorting stimuli into different categories) could be used to engage semantic processing. In addition to standardizing processing during encoding, the specific protocol employed during the retention interval could have important implications, potentially inducing retroactive memory interference [89]. Studies exploring long-term memory effects after a 24-hour delay should carefully consider the instructions that are given to participants as to their behaviors during that time period. At this point in time, additional research is needed to identify the optimal, noninterfering tasks to occur during the retention interval. 

Further, and as discussed elsewhere [33], careful consideration should be placed on the type of control task (e.g., disengagement, cognitive engagement, and active) utilized in exercise–memory research. Relatedly, sham exercise conditions (e.g., exercise with no load and stretching) could also be considered to help isolate the important exercise-related characteristics thought to enhance memory. Importantly, researchers should ensure that the exercise group and the control group only differ in the exercise being conducted. In other words, if the control group is watching a movie, the exercise group should also watch a movie during exercise. If the exercise group is sitting on a bicycle to exercise, the control group should also be sitting on a bicycle. Taking steps such as these to ensure that the protocol is administered cleanly and consistently across treatment groups or conditions, while also ensuring that the only difference between conditions is the independent variable, will contribute to more reliable and robust results in the future. Researchers should also carefully consider whether to implement exercise as a between- or within-subjects factor and whether memory assessments should occur after exercise or both before and after exercise [33]. Although within-subject, crossover, and pretest–posttest designs may yield stronger statistical power and inference for causation, there may be situations when such a design is potentially less optimal. For example, post-exercise memory assessments may be susceptible to proactive interference effects from pre-exercise assessments. Further, within-subject evaluations across multiple intensities and retention intervals may place an undue burden on participants, potentially inducing fatigue/apathy that may impact the consistency of results across conditions. Additionally, repeated exposure to the same type of task may induce learning effects by participants optimizing their strategy/processes for performing the task; this underscores the importance of incorporating an adequate familiarization session of the learning/memory task prior to the start of the study. Finally, when utilizing different test lists across conditions, it would be sensible to a priori psychometrically evaluate the tests (e.g., evaluate item difficulty, item discrimination, item determination, and test equivalency). However, when these concerns can be mitigated, within-subject designs may be optimal.

When evaluating the effects of acute exercise on long-term memory, the influence of sleep, which plays an important role not only in memory consolidation but, also, in encoding, should be considered. Sleep plays an important role in the consolidation of memory, as slow oscillations during slow-wave sleep (in the neocortex) drive the repeated reactivation of hippocampal memory representations during sharp-wave ripples; in contrast to slow-wave sleep, which helps consolidate non-emotional declarative memories, rapid eye movement (REM) sleep appears to consolidate emotional memories, although the role of this phase of sleep in memory is less clear [90]. Additionally, slow-wave sleep helps to downscale the strength of the synapses that were potentiated during encoding the information when previously awake, with this synaptic downscaling helping to ease the encoding of new information during subsequent wakefulness [90]. Notably, exercise prior to encoding, coupled with a post-encoding period of sleep, has been shown to be more effective in enhancing long-term memory when compared to no exercise or no sleep [91]. In addition to post-encoding sleep, research also demonstrates that exercise prior to encoding, coupled with a brief period of post-encoding mindfulness meditation during the consolidation period, is more effective in enhancing long-term memory when compared to exercise alone [92]. Together, these findings highlight that post-encoding behaviors, such as sleep and mindfulness meditation, may play a critical role in augmenting the exercise-induced consolidation of long-term memories. 

In addition to the aforementioned protocol characteristics, such as the method used to prescribe the exercise intensity, memory protocol, and behaviors (e.g., physical activity, sleep) occurring during the memory consolidation period, efforts should be made to not only standardize but, also, document other important aspects of the study, such as the time of day of the assessments and blinding the data analyst and/or the researcher administering the memory task. Lastly, it would be worthwhile to carefully consider (or reconsider) how select exclusionary criteria influences not only external but, also, internal validity. For example, it is common for studies to have participants refrain from caffeine consumption for an extended period of time (e.g., 3-6 h) before the session because of the potential ergogenic effects of caffeine. However, does this potential ergogenic aid of caffeine outweigh the potential negative cognitive effects of caffeine withdrawal for a regular coffee drinker? Further, studies often only include right-hand-dominant individuals in the study, as left-hand dominance, or, more accurately, mixed-handed individuals, tend to demonstrate superior memory performances [93]. Notably, there are many other individual characteristics (e.g., biological sex, and baseline cognitive performance) that are also associated with higher memory performance and that are not typically considered as exclusionary criteria. Additionally, it is unclear if handedness or any of these other individual characteristics actually influence the effects of exercise on memory (i.e., we do not know if these variables exert a main effect on memory performance or moderating effects on the influence of exercise on memory performance) [94]. As such, it may be more informative to consider some of these factors as potential moderators, as opposed to exclusionary criteria. 

***Chronic Exercise***. Various moderators of the chronic exercise–memory relationship have been discussed elsewhere [15]. For example, at this point, evidence suggests that chronic exercise may have larger effects on verbal–auditory and procedural memory. Chronic exercise involving walking and cycling appears to optimize short-term memory, whereas chronic exercise modality seems to negligibly impact the observed benefits for long-term memory. Long-term exercise programs lasting at least 6 months appear to be optimal in enhancing short-term memory, whereas the length of the program does not appear to influence the benefits observed for long-term memory [15]. In a recent meta-analysis focusing exclusively on working memory [26], age moderated the effects of chronic exercise on working memory, with larger exercise-related improvements in working memory occurring as age increases. 

Etnier et al. [25] conducted the first meta-analysis suggesting that the strength of benefits of acute and chronic exercise could depend on age group, with the largest effects found for middle-aged adults (aged 45–60 years). Indeed, subsequent meta-analyses have suggested the strength of benefits for chronic exercise training on memory are stronger for middle-aged and older adults [95] (declarative memory ES = 0.36, working memory ES = 0.29) than when young adults are also considered (declarative memory ES = 0.13, working memory ES = 0.03) [96]. Rathore and Lom [26] showed that chronic exercise was effective in enhancing short-term and working memory performances with a more pronounced effect for older adults. Overall, the results from these meta-analyses suggest that age moderates the chronic exercise effects on memory, with more pronounced benefits among middle-aged or older adults. The results from prospective study designs in late adulthood further support the plausibility that physical activity in late adulthood can slow memory loss over years to come, a benefit that appears stronger for episodic and working memory compared to semantic memory [97]. 

Additional research, including a wide variety of cardiorespiratory fitness levels among participants, is needed to better inform our understanding of how aspects of cardiorespiratory fitness may moderate the effects of chronic exercise on memory [15]. Although not supported by research evaluating overall cognition [98], there is some evidence that improvements in long-term memory [99,100], but not spatial working memory [101], from aerobic training are positively correlated with training-related changes in cardiorespiratory fitness among older adults. This is convergent with evidence from cross-sectional studies suggesting that cardiorespiratory fitness in older adults is more strongly related to hippocampal-based learning [102] and connectivity in the brain [103] than physical activity. 

Together, these studies suggest that the extent to which participants improve cardiorespiratory fitness from physical activity or training could play an important role in how much memory benefit is seen, and this effect may vary by memory type. The mechanistic nature of how fitness moderates, or possibly mediates, training-induced changes in different memory types and processes will benefit from future research considering the distinct components of fitness and memory within the same study. Importantly, researchers should ensure they are employing the correct analyses when considering interindividual differences in the cardiorespiratory response to the exercise intervention [104,105,106,107].

## 6. Suggestions for Future Research 

***Moderators.*** As described above, various factors may moderate the effects of exercise on memory function. Continued comprehensive research is needed to further improve our understanding of how the aforementioned factors (e.g., dimensions related to the individual, exercise protocol, and memory task) influence the exercise–memory relationship. Current meta-analyses often include a small number of studies in these moderation analyses, and a limited number of effect sizes coupled with grouping few studies may lead to misinterpretation. For example, when combining relatively few studies to evaluate if exercise intensity moderates the exercise–memory relationship, studies may differ based on the cognitive task or biological sex proportion, potentially confounding the moderation analysis. As such, empirical studies should be specifically designed to evaluate these potential moderators. We also need a better understanding of why these parameters play a moderating role in the exercise–memory relationship, which requires empirical evaluation. 

***Mechanisms.*** The field would also benefit from additional mechanistic studies in humans, evaluating the psychological and neurophysiological mechanisms through which acute and chronic exercise influence memory. Psychology and cognitive neuroscience are especially well-positioned to test predictions about the mechanisms through which acute and chronic exercise affect distinct human memory systems and processes. When appropriate, the integration of these disciplines with exercise science may be useful in comprehensively evaluating the plausible underlying mechanisms. For example, and although findings are mixed for declarative memory [108,109,110,111], exercise has been shown to improve procedural memory performance by attenuating memory interference [112,113,114], which could be explored through the lens of psychology and cognitive neuroscience [110,115].

As stated previously, exercise may influence memory through distinct mechanisms based on the memory phase (e.g., encoding, consolidation, or retrieval), which should be carefully considered in future mechanistic work. Additionally, the evaluated theoretical mechanisms may require a more fine-grained evaluation of the memory task. As an example, if the goal is to evaluate if exercise improves memory through representational processes (although speculative, exercise may improve memory via representational processes, as embodied or enactment movement may help to increase a mental representation of the stimuli; further, increasing levels of exercise intensity may increasingly activate the hippocampus, a critical subcortical structure involved in representational processes), the degree of representational dependency may vary considerably based on the task, increasing from recall to simple yes/no recognition tasks on stimuli with varying levels of representational similarity to paired-associative and cued-recall tasks. Further, specific memory outcomes (e.g., implicit, neutral episodic, and emotional episodic) and types of processing (e.g., incidental and intentional) may involve unique brain regions and underlying processes and, thus, may require the evaluation of specific mechanisms. Thus, careful consideration of the memory task, memory outcome, and type of processing will be needed when evaluating the theoretical mechanisms through which exercise may improve memory. 

***Interacting Memory Systems***. We also encourage future work in less explored areas. An interesting area to consider in future research is whether the potential interacting effects of different memory systems can moderate the effects of acute exercise on long-term memory [112]. For example, acute exercise has been shown to enhance short-term working memory capacity and executive function [14]. These improvements may play important roles in facilitating the effects of exercise on long-term memory. For example, improvements in different aspects of prefrontal cortex function, a critical brain region involved in working memory and executive function [116], is involved in various aspects of long-term memory. During encoding, the dorsolateral prefrontal cortex may help organize and update information that is actively maintained in the ventrolateral prefrontal cortex. During retrieval, the ventrolateral prefrontal cortex may help cue information to be recalled from memory, with the dorsolateral prefrontal cortex helping to monitor and evaluate this information [1]. Given the proposed interactions of working and long-term memory, it is plausible that the acute exercise effects on working memory systems [46,117] may play a role in encoding and retrieval processes during tests of long-term memory. However, whether and how exercise affects memory system functions per se, or the interaction of memory and executive systems or both, remains an important question for future research. 

***Phases of Memory.*** In addition to evaluating whether acute exercise-induced changes in working memory predict improvements in long-term memory functions, we encourage future research to investigate if engaging in exercise during separate phases of memory (e.g., before and during encoding and during consolidation) can have a synergistic effect on long-term memory. Loprinzi et al. [118] initially tested this by having young adults engage in a 15-min bout of moderate-intensity acute exercise both before memory encoding and during consolidation, compared to a separate session where exercise occurred only before encoding. The long-term memory performance was similar across conditions, suggesting that engaging in exercise during both memory phases was not greater in improving memory than when exercise was performed in a single memory phase. Loprinzi et al. [29] followed this up with a separate experiment but, instead, used a high-intensity bout of acute exercise, ultimately showing that exercising during both phases was better at enhancing long-term memory when compared to exercising during a single memory phase. Similar findings were also demonstrated by Slutsky-Ganesh, Etnier and Labban [22]. Despite these studies, additional work is needed in this area. For example, if exercise does additively influence memory when occurring in multiple phases (e.g., before encoding and during consolidation), it is unclear as to the optimal time within each phase that these exercise bouts should occur (e.g., before encoding and early consolidation or before encoding and late consolidation) [29].

Both acute and chronic exercise may have beneficial effects on improving memory functions. It may be beneficial, however, to not consider them in isolation. When evaluating the effects of chronic exercise on memory, the placement of the last bout of acute exercise, in reference to the final memory assessment, should be carefully considered. For example, Hopkins et al. [58] had participants complete an object recognition task (exposure and recognition in the same session) on two separate visits separated by four weeks. Between the two visits, participants were randomized into a control or exercise training group for four weeks, with the last bout of exercise either occurring or not occurring on the same day as the second visit. The results showed that object recognition performance improved with four weeks of exercise training, but this only occurred in the group that exercised on the final day of testing. This suggests that some of the observed effects of chronic exercise on memory may be influenced by the acute effects of exercise or that acute exercise has a priming effect on the chronic effects of exercise [119]. 

Another interesting observation of the potential interactive effects of acute and chronic exercise comes from recent research demonstrating that changes in working memory performance in response to moderate-intensity acute exercise may predict improvements in working memory from chronic exercise [46]. Thus, the intensity-dependent memorial response to acute exercise may serve as a marker of the potential for adaptive long-term effects from chronic exercise. This may provide useful insight into which individuals may garner the greatest memory benefits from chronic exercise. Future works should continue exploring this exciting possibility. 

## 7. Conclusions

In conclusion, accumulating evidence suggests that both acute and chronic exercise can improve the performance of various memory systems. However, this effect appears to be influenced by a multitude of factors related to, for example, the individual, exercise protocol, memory type, and other characteristics of the study design. At this time, relatively few empirical studies have been specifically designed to examine the effects of these moderating variables, and studies have been largely focused on young college-aged adults with a much smaller body of literature addressing other age groups and even fewer studies adopting a lifespan approach. In addition to needing more research on individual moderators, future research should consider that some of these moderators may interact with each other to uniquely predict the benefits obtained. In addition to research-exploring moderators, a focus on mechanisms is also warranted. Various mechanisms at multiple levels (e.g., cellular, molecular, and systems) have been discussed as potential explanations as to how acute and chronic exercise can influence memory. However, the majority of these mechanisms have only been examined in animal models, necessitating the need for more mechanistic work in humans. We also highlight the need for future research to examine the interactive effects of acute and chronic exercise on memory. Potential questions to ask include whether chronic exercise behavior influences the memory responses to acute exercise and whether the memory response to acute exercise predicts memory adaptations from chronic exercise. We hope these recommendations will help move the field forward, and we look forward to the advances and new knowledge that are inevitable in this growing and exciting field.
